# ReMODE: a deep learning-based web server for target-specific drug design

**DOI:** 10.1186/s13321-022-00665-w

**Published:** 2022-12-12

**Authors:** Mingyang Wang, Jike Wang, Gaoqi Weng, Yu Kang, Peichen Pan, Dan Li, Yafeng Deng, Honglin Li, Chang-Yu Hsieh, Tingjun Hou

**Affiliations:** 1 Innovation Institute for Artificial Intelligence in Medicine of Zhejiang University, College of Pharmaceutical Sciences and Cancer Center, Zhejiang University, Hangzhou, 310058 Zhejiang People’s Republic of China; 2 CarbonSilicon AI Technology Co., Ltd, Hangzhou, 310018 Zhejiang People’s Republic of China; 3 Shanghai Key Laboratory of New Drug Design, School of Pharmacy, East China University of Science & Technology, Shanghai, 200237 People’s Republic of China

**Keywords:** Deep learning, De novo drug design, Molecular generation, Adversarial autoencoders, Transfer learning, Artificial intelligence

## Abstract

Deep learning (DL) and machine learning contribute significantly to basic biology research and drug discovery in the past few decades. Recent advances in DL-based generative models have led to superior developments in de novo drug design. However, data availability, deep data processing, and the lack of user-friendly DL tools and interfaces make it difficult to apply these DL techniques to drug design. We hereby present ReMODE (Receptor-based MOlecular DEsign), a new web server based on DL algorithm for target-specific ligand design, which integrates different functional modules to enable users to develop customizable drug design tasks. As designed, the ReMODE sever can construct the target-specific tasks toward the protein targets selected by users. Meanwhile, the server also provides some extensions: users can optimize the drug-likeness or synthetic accessibility of the generated molecules, and control other physicochemical properties; users can also choose a sub-structure/scaffold as a starting point for fragment-based drug design. The ReMODE server also enables users to optimize the pharmacophore matching and docking conformations of the generated molecules. We believe that the ReMODE server will benefit researchers for drug discovery. ReMODE is publicly available at http://cadd.zju.edu.cn/relation/remode/.

**Graphical Abstract:**

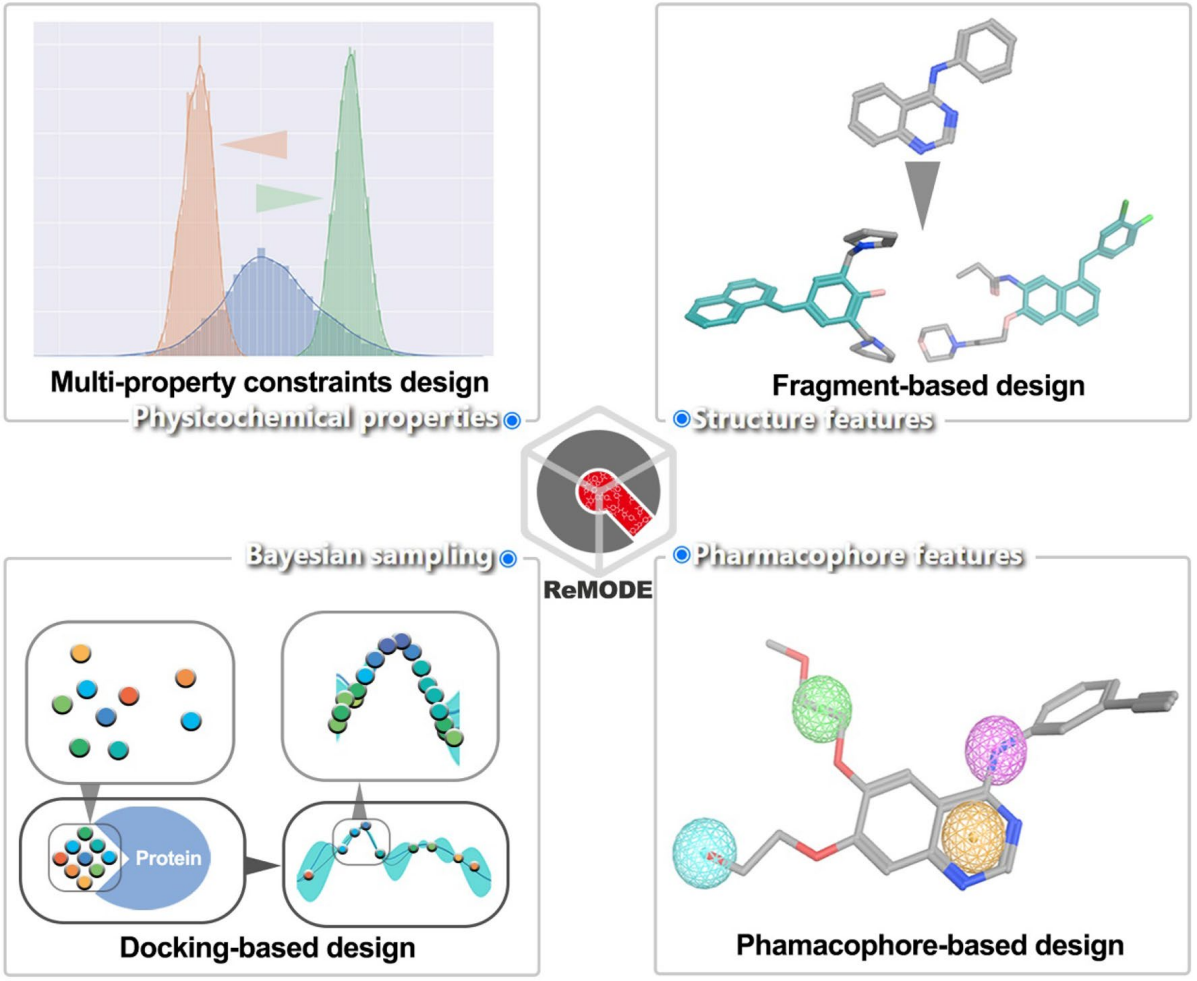

**Supplementary Information:**

The online version contains supplementary material available at 10.1186/s13321-022-00665-w.

## Introduction

In the past few decades, the cost of a new drug from research and development to the market was estimated to be between 314 million and 2.8 billion US dollars, which takes more than 10 years on average [1, 2]. It is estimated that only 0.1% of candidates make it through preclinical testing to human testing and just one-fifth of those reach the market [3]. Therefore, there is an urgent need to supplement the pipelines of drug discovery with enough drug candidates [4, 5]. Virtual screening (VS) approaches have been widely used to mine novel chemical entities from in-house or commercial compound libraries based on either molecular docking toward a known target structure or similarity to known active compounds. Although VS-based drug discovery approaches can identify active compounds, they are limited by the chemical libraries used.

Contrary to VS, de novo drug design can create molecules that do not exist in the known compound libraries. De novo drug design approaches usually create molecule structures from given objectives and have the advantage of exploring a much wider chemical space beyond existing compound libraries. During the last decades, various traditional de novo drug design approaches have been developed, and they can be divided into two categories: structure-based [6–11] and ligand-based [12–14]. In structure-based approaches, the three-dimensional (3D) structures of targets are utilized to infer the information related to important protein–ligand interactions [9, 15–18], and have been emerged as valuable tools in the design of novel inhibitors toward important targets [19–22]. Ligand-based approaches usually use the collections of compounds that are known to bind to specific targets to guide the generation of novel structures [13, 14, 23–26].

Recently, machine learning (ML) methods, especially DL methods, have brought many new breakthroughs to the field of de novo drug design, and numerous de novo drug design methods based on different DL architectures have been developed [27, 28], such as recurrent neural networks (RNN) [29–32], variational autoencoders (VAE) [33–35], generative adversarial networks (GAN) [36] and reinforcement learning (RL) [37–39]. Nevertheless, unlike the majority of traditional de novo design methods, most of these DL-based generative algorithms are ligand-centric and represent molecules as either 1D SMILES strings or 2D molecular graphs. A clear shortcoming of these representations is that the generative models cannot capture the critical conformation of 3D molecular geometry toward a specific protein target, and the generated molecules may be only subject to the ligands in the training set. Therefore, these methods are not suitable for the design of novel chemical entities in a target-specific task.

To generate active chemical entities, a feasible strategy is to incorporate the 3D molecular geometry into the generative model. There has been a series of studies [34, 40–49] attempting to incorporate 3D molecular geometry into DL-based generative architectures. These models typically transform molecules into 3D conceptual graphs (such as voxels [49], mesh shapes [34, 40] or 3D molecular graphs [43, 45–47]) of molecules, and use pharmacophore matching coefficients or docking scores to guide the process of molecule generation. However, the application of these DL algorithms is difficult due to the data availability, computational resource cost, open-source nature of the code, or lack of user-friendly tools.

Based on these premises, we developed the ReMODE server, the first web server to perform the target-specific tasks with our newly-developed DL-based algorithm RELATION [49]. RELATION is trained with two sets of 3D grids of training data: ligand-only (source dataset) and ligand-target binding complexes (target dataset). The BiTL (bidirectional transfer learning) was specifically designed to capture the subtle relations between these two datasets, and extract and transfer the desired geometric features of strongly bounded ligands from the ligand-target binding complexes to a ligand-only latent space for efficient molecular generation.

We have trained the RELATION models using the data of 23 targets, and users can select a target in the list to create the generative task and generate molecules rapidly in the ReMODE server. However, in the practical application of a generative model, these randomly generated molecular set sometimes cannot meet the needs of drug design, and researchers prefer to control the different properties of the molecular set and create some customizable tasks. For this purpose, the conditional models of all protein kinases were also trained and integrated into the ReMODE server, which allows users to create the multi-property constraints task and fragment-based design task in the “Physicochemical properties” and “Structure features” modules. Other key modules of ReMODE are the “Pharmacophore features” and “Bayesian Optimization”, and the use of these two modules will generate molecules with favorable pharmacophore matching scores and docking conformations, and these molecules will have greater potential as drug candidates.

To sum up, with the multifunctional modules mentioned above, our ReMODE server is believed to have a greater capacity to assist medicinal chemists in performing customizable de novo drug design and accelerating the drug research and discovery process. ReMODE is implemented as a publicly available web server with a user-friendly interface and can be freely accessed at http://cadd.zju.edu.cn/relation/remode/.

## Materials and methods

### The generative model in ReMODE

The framework of RELATION model [49] was selected as the main backend of the ReMODE server. The RELATION network [49] consists of two parts: the private and shared encoders based on 3D-convolutional neural network (CNN); the captioning decoder based on captioning long short-term memory (LSTM). The RELATION model was trained with the ZINC dataset and protein–ligand complex dataset simultaneously, the ZINC dataset contains massive structural storage of compounds, and the protein–ligand complex dataset contains the binding information of protein–ligand complexes. If only a large-scale ZINC set is used to train the generative model, the generated molecules cannot focus on our desirable properties. And for the protein–ligand complex dataset, the number is too few to train a valid generative model. So the transfer learning was used in the training of the generative model, and the ZINC dataset and the ligand–protein complexes were set as the source domain and target domain, respectively [50–53]. Herein we adopted DSN (Domain Separation Networks) [54], a bidirectional transfer learning method based on the DL framework, in which the latent space contains not only the shared embedding between the two domains but also the unique features. That is to say, we intended to bidirectionally transfer the characteristics of ligands and ligand–protein complexes to a latent space for generation. This bidirectionally transfer endows ReMODE to generate molecules with both novelty and 3D geometry features [49].

### Framework of ReMODE

To extend or build upon existing server functionality, we modified the basic framework of the RELATION model. To enable the ReMODE server to generate molecules with desirable properties and fragment/scaffold/structure, CVAE (Conditional variational autoencoder) was utilized to control multiple physicochemical properties and structural features by imposing them on a latent space. In Bayesian Sampling module, AutoDock Vina [55] docking scores were selected as the black-box objective function of BO (Bayesian Optimization) process. In BO process, sparse GP was used for modelling the surrogate model, expected improvement was used as acquisition function [33, 56]. And for pharmacophore modules, LigandScout 4.4.7 [57, 58] was used as the scoring function. The pharmacophore properties were constructed from the LigandScout Pharmacophore Database. The pharmacophore properties in CVAE was set to ‘Relative Pharmacophore-Fit’, and the maximum number of omitted features was set to ‘1’ for the iscreen tool provided with LigandScout 4.7.7. The hyper-parameters and framework details available in ReMODE were recorded in supplementary data and listed in Additional file 1: Tables S1–S4.

### Data processing

The source dataset was prepared based on the ZINC Clean Lead database [59, 60]. We removed the molecules containing charged atoms or atoms besides carbon, nitrogen, oxygen, sulfur, fluorine, chlorine, bromine and hydrogen. Then molecules with molecular weight (MW) ranging from 200 to 600 and predicted logP from -2 to 6 were selected as the source dataset. Furthermore, 23 protein–ligand complexes and protein kinase inhibitors (PKIs) set and were (IC_50_ < 50 nM) collected from BindingDB [61], ChEMBL [62] and PDB data bank [63] were used as the target dataset of ReMODE. Same as our past research [49], the two datasets were represented as the 3D-grid format, with each atom in the 3D-grid described by 19 physicochemical properties (a 4D tensor for network). These data can be downloaded from our server (http://cadd.zju.edu.cn/relation/remode/doc/). We also built a drug-target network between target and PKIs [64, 65], and therefore users can model the target-specific drug design task from the perspective of network [66–69].

### Web server implementation

The ReMODE server is a publicly accessible server, which could be accessed through a web browser, and the backend was developed using the Python web framework of Django and deployed on an elastic compute service from Aliyun running an Ubuntu Linux system. The web access was enabled via the Nginx web server and the interactions between Django and proxy server were supported by uwsgi. This application was developed based on the Model-View-Template (MVT) framework. The model layer maps the business objects to the database objects, and the storage and management of the submitted job data were implemented by SQLite3. The view layer is a business logic layer, responsible for performing the access to the ReMODE models, delivering the generated molecules to be shown on the template layer. The template layer provides the visualization of results, page rendering, integration of documentation, etc. The downloaded files, analysis result and pre-trained models were stored in the server. PyTorch was used in the construction of the ReMODE model. Additionally, the RDKit package was employed to provide various cheminformatics support. The server has been successfully tested on the recent version of Microsoft Edge, Google Chrome and Apple Safari.

## Results and discussion

**Fig. 1:**
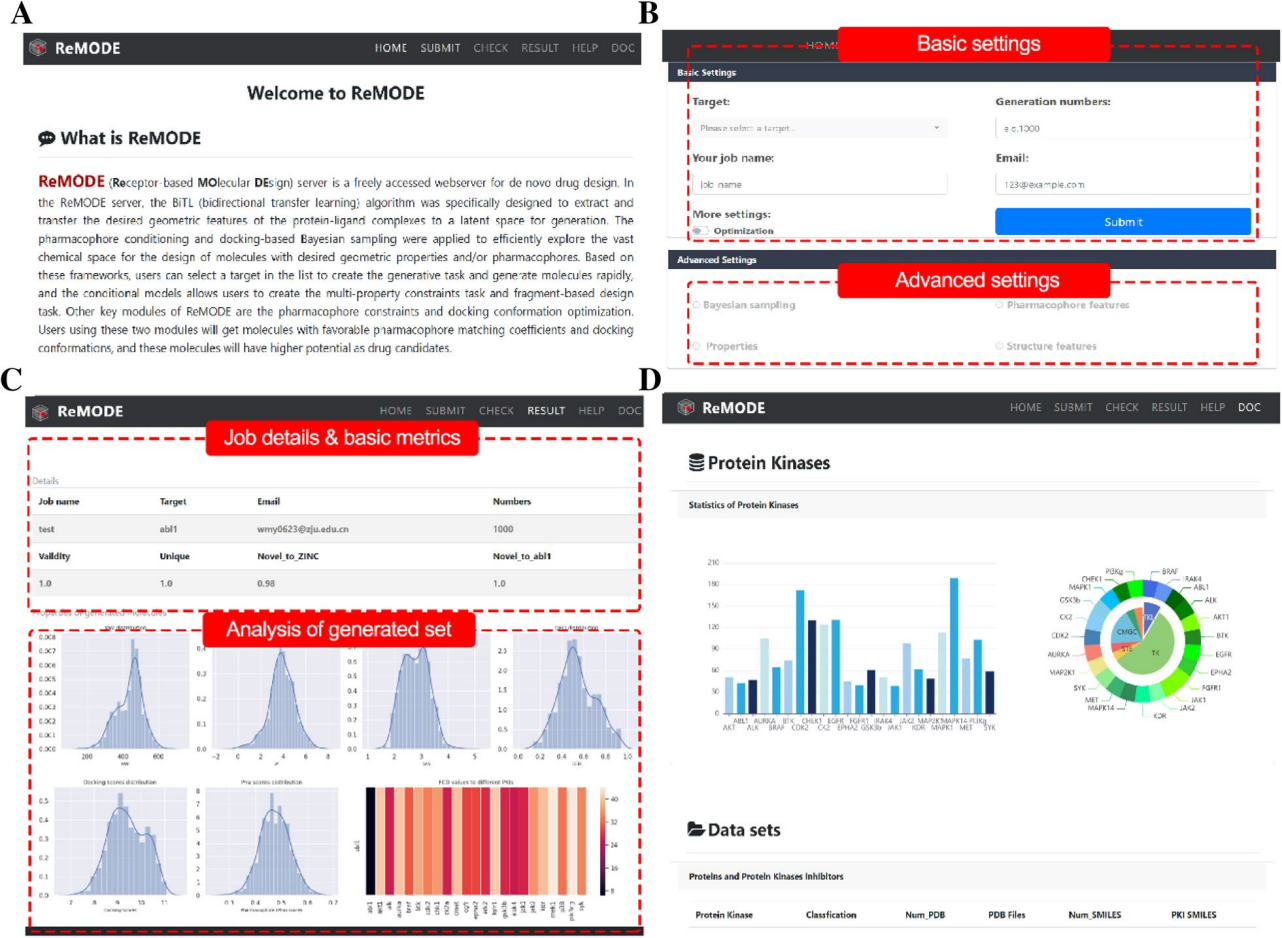
Detailed view of the ReMODE server: **A** Introduction of the protein kinases profiling and DL-based algorithms in the ReMODE server; **B** Details of the target-specific creation interface with various functional modules; **C** Example of an analysis result of generated molecules; **D** Details page for the different dataset used in model training, and the download links are also provided

As designed, ReMODE allows users to perform customizable target-specific design tasks, as described below. The key examples and step-by-step instructions on how to create a task in the ReMODE server are provided in the ‘Help’ section of the website (available at: http://cadd.zju.edu.cn/relation/remode/help/). The algorithms of the different modules have been documented in Additional file 1. The detailed view of the ReMODE server is shown in Fig. 1.

### Creation of unconditional generation task

In unconditional generation task, all the protein targets in list have a pretrained generative model. If the switch ‘Optimization’ is off, users can select a target from the list, then enter the task name, email address, and the number of molecules to be generated quickly to quickly generate the target-focused molecules.

### Creation of conditional generation task

The ReMODE server allows the user to create conditional target-specific tasks. In conditional tasks, we made CVAE (conditional Variational Auto-Encoder) framework of ReMODE more suitable for multi-constraint control. CVAE can incorporate the information of molecular properties in the encoding process and manipulate them in the decoding process. Users can turn on the switch ‘Optimization’ to select one of the conditional modules from ‘Physicochemical properties’, ‘Pharmacophore features’ or ‘Structure features’ options.

In the “Physicochemical properties” module, the framework was used to generate target-focused molecules satisfying four target properties at the same time: MW (molecular weight), logP (partition coefficient), QED (quantitative estimate of drug-likeness), and SA (synthetic accessibility). This module allows users to get a number of molecules with the specific values of MW and LogP within given ranges, and with the optimized values of QED and MW. It was also possible to adjust a single target property without changing the others.

In the “Pharmacophore features” module, users can generate molecules with pharmacophore constraints. The pharmacophore properties in CVAE was set to ‘Relative Pharmacophore-Fit’ to a pharmacophore model, and the maximum number of omitted features is set to ‘1’ for the *iscreen* tool in LigandScout [57, 58]. The pharmacophore models used for each were collected from the LigandScout Pharmacophore Database (http://www.inteligand.com/pharmdb/), which is based on many years of experience in pharmacophore creation and has been assembled manually and quality checked carefully.

The “Structure features” module is designed to perform fragment-based generation, and it allows users to upload a fragment/scaffold/sub-structures as the starting points for creating fragment-based design task. The uploaded SMILES string is used as the caption data in the latent space, and collected the latent points within 5 Euclidean distances to the caption data. These latent points contain the 3D geometry features of protein–ligand complex and can be decoded to molecules with high structural similarity to the uploaded structure.

### Creation of Bayesian sampling generation task

The ‘Bayesian Optimization’ module allows users to generate the target-focused molecules with high docking scores and favorable docking conformations. Bayesian optimization is performed in the latent space to find molecules that score highly under a specified Autodock Vina docking function, and the detailed procedure of Bayesian Optimization is recorded in Additional file 1: Table S3.

### Model validation

In this section, we conducted a comprehensive evaluation of the generated molecules from each selected protein kinase in list to examine the performance of different modules (in the “Physicochemical properties” modules, the “MW” was set to 250 to 750, the “logP” was set to − 2 to 8, and the “QED” and “SA” were set to off) in the ReMODE server.

We first tested some basic metrics of different modules, such as the validity, novelty, and uniqueness of the generated molecules. The results in Additional file 1: Figure S1 show that the introduction of CVAE will lead to the decrease of validity in some modules (“Physicochemical properties”, “Structure features” and “Pharmacophore features”). Therefore, in order to improve user experience, when the users select these modules, the server will call the background generative model and will not stop running until the number of valid molecules required by users is generated.

**Fig. 2:**
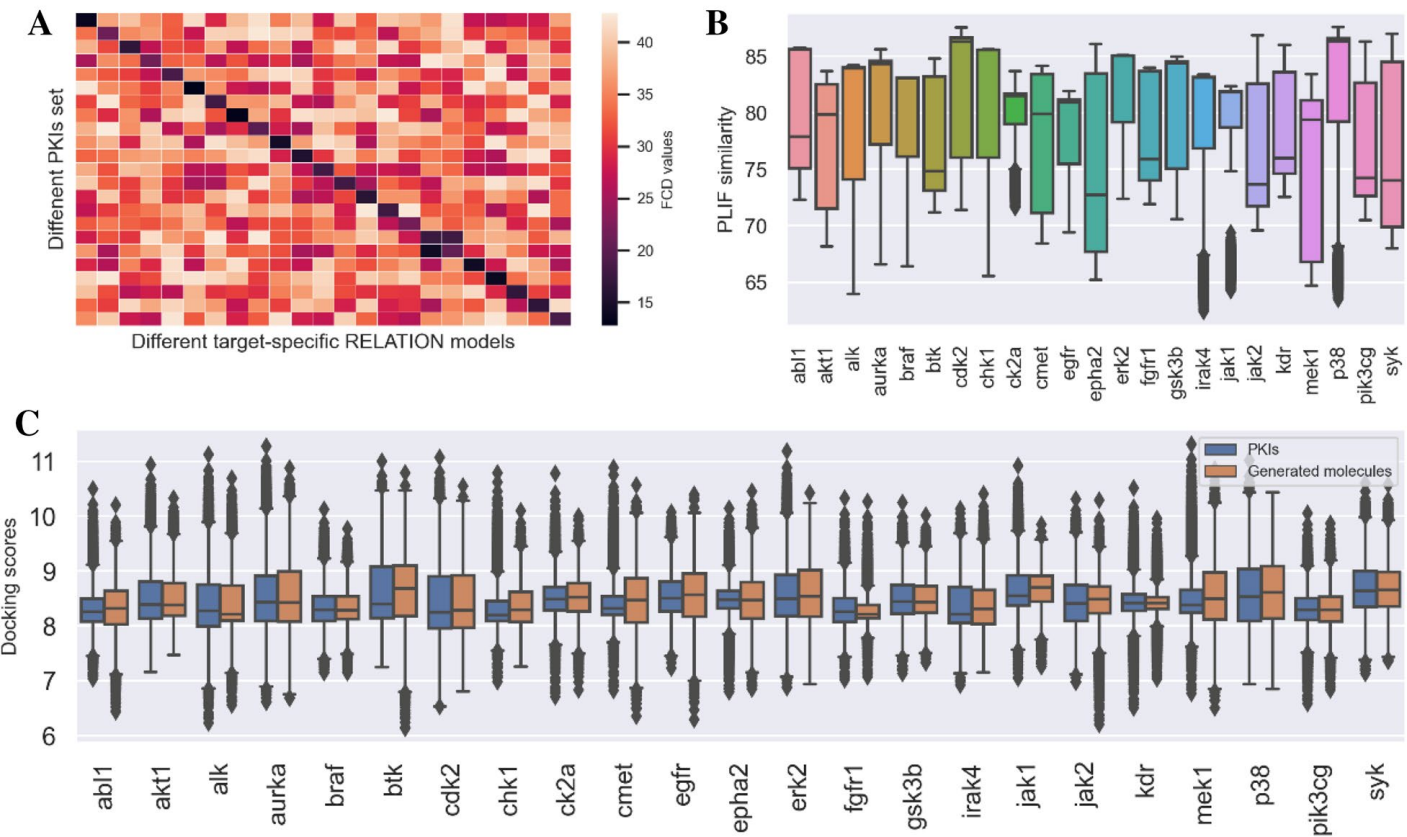
Statistics of the generated molecules for 23 different target-specific tasks. **A** The FCD values of the generated molecules for the 23 protein targets (‘Bayesian optimization’ module), and the order and information of the targets in the horizontal and vertical coordinates are the same as those in the horizontal coordinates of **B**, **C**. **B** The average SNN of PLIF between the generated molecules and the PKIs set (‘Bayesian optimization’ module). **C** The docking score distribution of the generated molecules and the PKIs set (‘Bayesian optimization’ module). The detailed evaluations of the other modules are available in Additional file 1

The main function of our ReMODE server is to perform the target-specific generation task for each selected target in list, so we need to determine whether the generated molecules are target-focused. If the molecular set generated from a selected target demonstrates a high biochemical similarity to the reported PKIs of this target, but low similarity to other targets, then the generative model can be considered to be highly target-focused. The FCD (Fréchet ChemNet Distance) was used to inspect whether the generated molecules are diverse and have similar chemical and biological properties compared with the PKIs dataset. From the heatmap of the five modules in Fig. 2A and Additional file 1: Figure S2, it can be clearly seen that the generated molecular set with more favorable FCD values to the inhibitors of the selected target than the other targets, indicating that the molecules generated by ReMODE are target-focused.

In addition to the target-focused properties of the generated molecules, the binding affinities of the generated molecules to the target should also be considered. The Autodock Vina scores and the similarity of PLIF (protein–ligand interaction fingerprint) were set as the metrics to preliminarily investigate the binding affinities of the generated molecules. As shown in Fig. 2C and Additional file 1: Figure S3, the docking scores of the generated molecules for each selected target show a similar distribution to those of the target PKIs. Moreover, the similarities of nearest neighbor (SNN) of PLIF between the generated molecules and PKIs were also calculated to inspect the conformations of the docking complexes. In Fig. 2B and Additional file 1: Figure S4, it can be found that the similarities of PLIF of the docking conformations between the generated molecules and the PKIs are higher than 70%. In summary, we can find that the molecular set generated for each selected target demonstrates favorable docking scores and docking conformations, and we may have higher probability to select candidates with high binding affinities.

Due to the special properties of the molecules generated by the modules “Pharmacophore features” and “Structure feature”, we evaluated the molecules generated by the two modules separately. In Additional file 1: Figure S5A, it can be found that the molecules generated by the “Pharmacophore features” module have higher pharmacophore scores than those generated by the others modules. This suggests that, by turning on the “Pharmacophore features” modules, the generated molecules can have enhanced matching to the preset pharmacophore models. To evaluate the “Structure feature” in fragment-based drug design, we randomly selected a molecule in each PKI dataset of 23 targets as the input structure, and investigated the SNN distribution between the generated molecules and the input structure. As shown in Additional file 1: Figure S5B, the average SNN between the generated molecules and the input structure is close to 0.7 for all 23 targets, indicating that the “Structure feature” module can achieve the fragment-based design according to an input structure.

### Case study of de novo design of EGFR inhibitors

The above “Model validation” section has given an overview of the overall performances of all the 23 targets. In this section, we choose EGFR (Epidermal growth factor receptor) [70] as a case study to show the performance of the different modules in the ReMODE server.

**Fig. 3:**
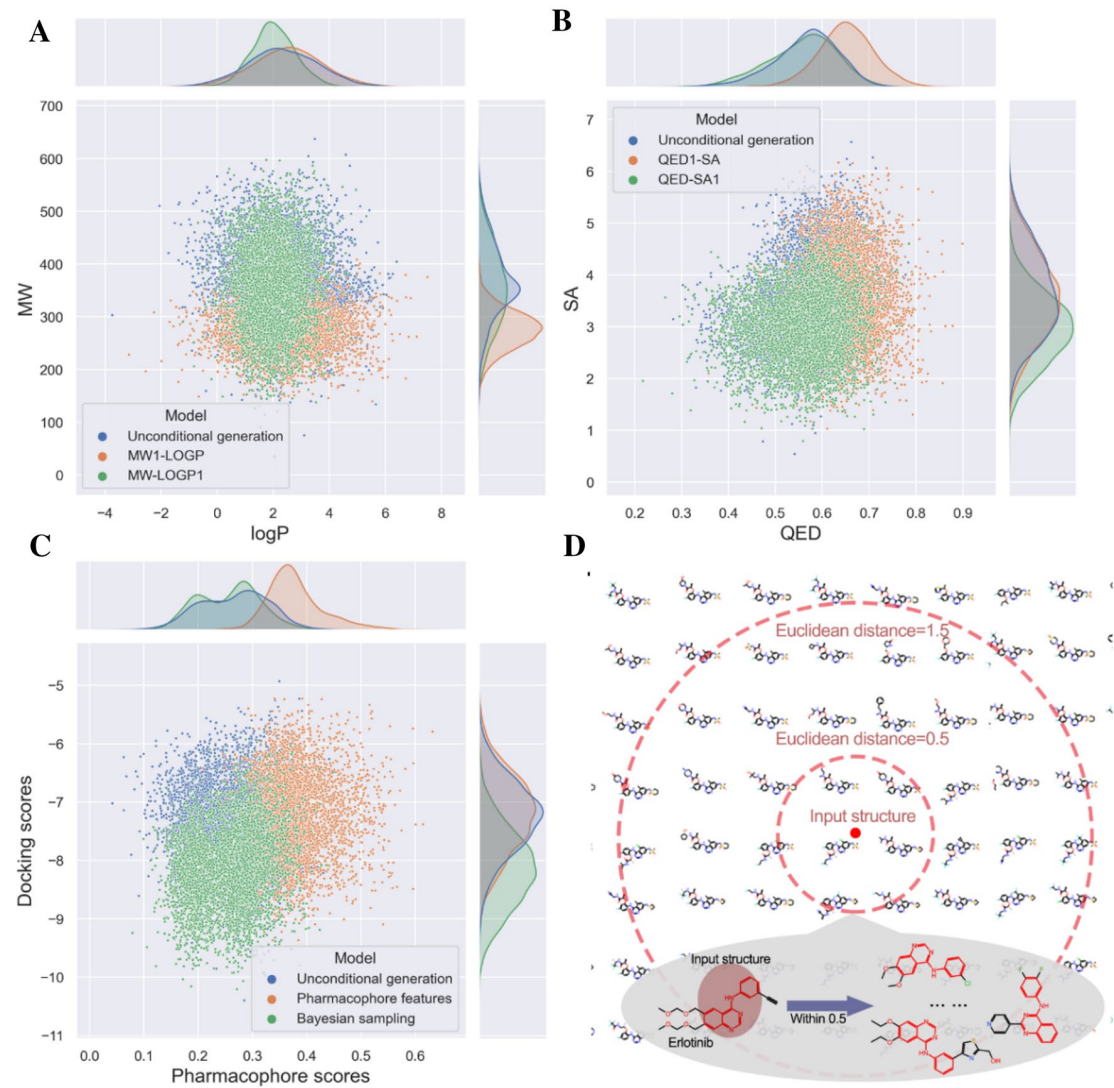
(**A-C**) The properties distribution of the 5000 valid molecules generated by the modules “Physicochemical properties” (**A**, **B**), “Pharmacophore features” (**C**), and “Bayesian sampling” (**C**) are compared with those generated by the unconditional generation task. **D** The scaffold of erlotinib was selected as the input molecule for the “Structure features” module to perform fragment-based design. Annotation: MW1-LOGP: the MW and logP range were set to 150–400 and -2–6, and no optimization for QED and SA; MW-LOGP: the MW and logP range were set to 200–600 and 0–4, and no optimization for QED and SA; QED1-SA: the MW and logP range were set to 200–600 and -2–6 and optimization for QED was turned on; QED-SA1: the MW and logP range were set to 200–600 and -2–6, and optimization for SA was turned on

With the introduction of different condition vectors (in our model, the customized properties of molecules we want to control were represented as the condition vectors) in the CVAE framework, the customized properties are directly involved in the encoder and decoder, and the sampling latent vector is composed of the customed properties and molecules. In this case study, the results of the multi-property constraint generation using “Physicochemical properties” and “Pharmacophore features” are shown in Fig. 3A–C. The QED, SA and pharmacophore score distributions of the generated molecules indicate that these modules in the ReMODE server are able to constrain the properties based on the customized property settings by users.

DL-based de novo drug design is usually applied in the initial stage of the discovery of novel drug candidates. Computational approaches such as molecular docking or other VS methods were normally used to select molecules with required properties from the generated compound library, and the top-ranked molecules were then selected for structural modification, synthesis and biological evaluation. The docking scores of the molecules toward EGFR are shown in Fig. 3C, where most molecules generated by the ‘Bayesian optimization’ module exhibit better docking scores of around − 9 to − 8 kcal/mol. However, the molecules generated by the other modules have the docking scores of around − 8 to − 7 kcal/mol, indicating that the molecules generated by the BO-based architecture are more likely to succeed in the following screening.

For the fragment-based generation in the “Structure features” module, the network of unconditional generation was selected as the basic architecture. Then, the input molecule (fragment/scaffold) will be decoded into the latent space as an anchor vector. Finally, a certain number (the number of generated molecules defined by users) of vectors are randomly sampled within a Euclidean distance of 5 around the anchor vector and decoded into a molecule set for users. The results in Fig. 3D and Additional file 1: Figure S3A can prove that our “Structure features” can carry out fragment-based drug design by setting a molecular structure as an anchor to discover compounds with the same substructure or high similarity in the chemical space.

**Fig. 4:**
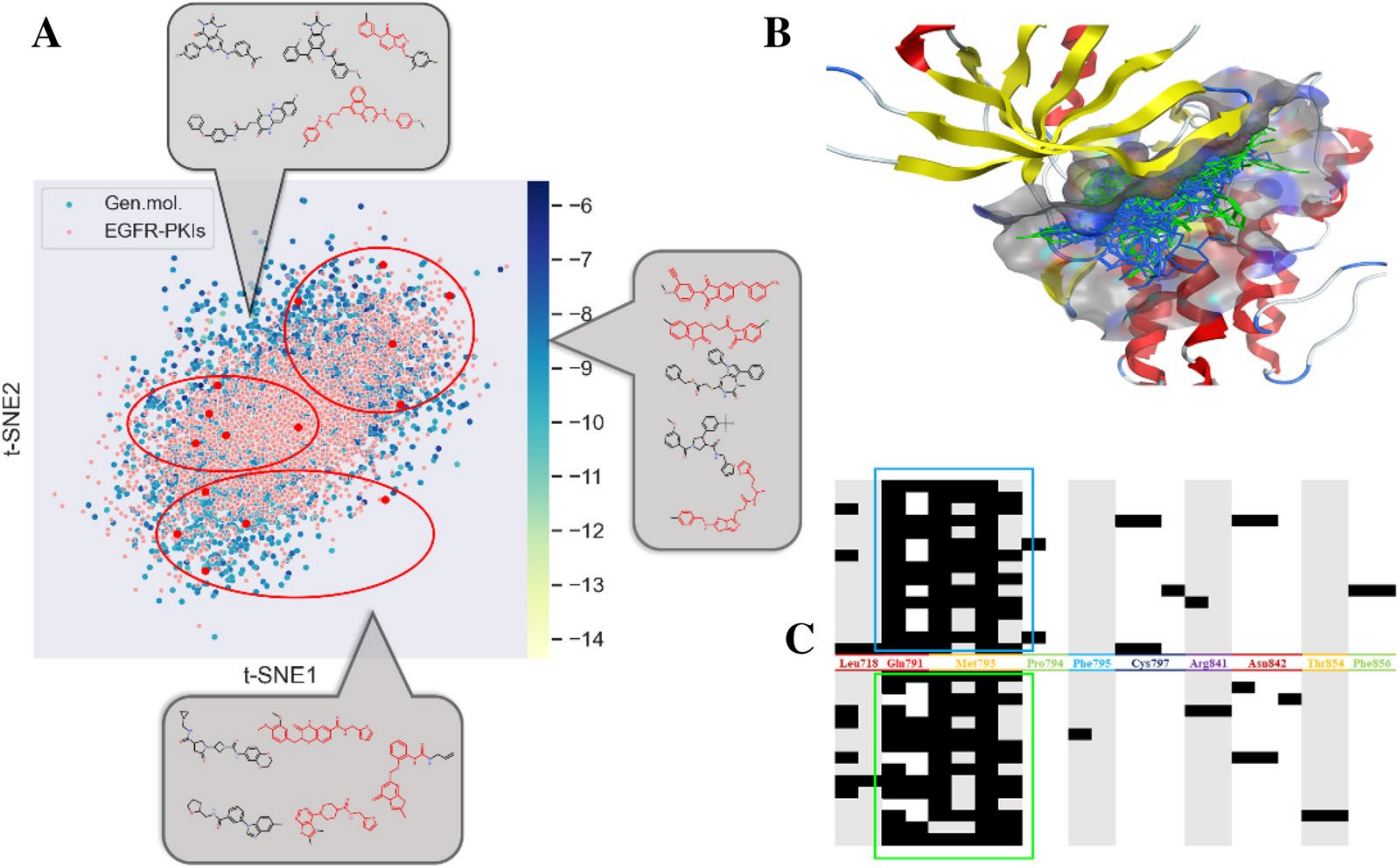
The analysis result of the generated molecules. **A** The t-SNE distributions of EGFR PKIs (orange) and generated molecules (blue, top 2000 molecules ranked by the docking scores, the lighter the color, the more negative the Vina docking score). The right points refer to the top 15 molecules ranked by the docking scores, the red part in these molecules are the active scaffolds reported (checked on SciFinder); **B** the docking conformation of these 15 molecules (green) and EGFR PKIs (blue, PDB IDs:4ZTQ, 4ZTR, 4ZTS, 5AAD, 5AAE, 5AAF, 4JBO, 4JBP, 4JBQ, 4DEA, 4DEB, and 4DED); **C** the protein–ligand interaction fingerprint of generated molecules (green) and EGFR PKIs (blue) to protein pocket

The 10,000 valid molecules generated by the ‘Bayesian optimization’ module were used for further assessment. In Fig. 4A, the t-SNE plot demonstrated that molecules generated by the ReMODE server have a great overlap distribution to EGFR TKIs in the chemical space. The binding patterns of the generated molecules and PKIs in the protein pockets are shown in Fig. 4B. The superposition in the protein pockets showed that the two molecular sets occupy a similar space of the binding pockets and have similar docking conformations. The hydrogen bond, ionic attraction, and surface contact that occur in the binding conformations are counted as protein–ligand fingerprints. In Fig. 4C, the generated molecules and compounds also have highly similar binding patterns in the pockets of EGFR.

## Conclusion

Although the powerful performance of DL technology in de novo drug design is widely recognized in both academia and industry, the satisfactory user-friendly tools are still limited. In current work, we presented ReMODE, a highly automated and efficient DL-based de novo drug design web server. The basic functions of the ReMODE sever can perform target-specific tasks for the 23 protein targets. Meanwhile, our ReMODE server also integrates four extended modules ‘Physicochemical properties’, ‘Structure features’, ‘Pharmacophore features’, and ‘Bayesian sampling’, and users can easily create customizable generation tasks such as multi-property optimization, fragment-based design, or pharmacophore constraints design via these modules. To some extent, ReMODE still has some limitations. Currently, ReMODE only supports protein target in list, and does not allow users to upload their own targets to generate target-focused molecules. Since the training ReMODE model requires a large number of protein–ligand complexes, it is not convenient to upload these data through the website. In the future, more advanced DL-based algorithms will be added to the ReMODE server, making it more versatile and user-friendly for users. ReMODE aims to be the first open accessible DL-based target-specific design platform and more functional modules will be implemented into this platform. We believe that our ReMODE web server would facilitate DL-based de novo drug development and benefit the discovery of novel bioactive candidates.

## Supplementary Information

**Additional file 1. **

## Data Availability

The web server is available at http://cadd.zju.edu.cn/relation/remode/. The data and source code are available at https://github.com/micahwang/ReMode/tree/master.

## References

[1] Wouters OJ, McKee M, Luyten J (2020). Estimated research and development investment needed to bring a new medicine to market, 2009–2018. JAMA, J Am Med Assoc.

[2] DiMasi JA, Grabowski HG, Hansen RW (2016). Innovation in the pharmaceutical industry: new estimates of R&D costs. J Health Econ.

[3] Dominguez LW, Willis JS (2020). Research and development costs of new drugs. J Am Chem Soc.

[4] Scotti L, Scotti MT (2020). Recent advancement in computer-aided drug design. Curr Pharm Des.

[5] Bhagat RT, Butle SR (2021). Drug repurposing: a review. J Pharm Res Int.

[6] Böhm H-J (1992). LUDI: rule-based automatic design of new substituents for enzyme inhibitor leads. J Comput Aided Mol Des.

[7] Clark DE, Frenkel D, Levy SA, Li J, Murray CW, Robson B, Waszkowycz B, Westhead DR (1995). PRO_LIGAND: An approach to de novo molecular design. 1. Application to the design of organic molecules. J Comput Aided Mol Des.

[8] Gillet VJ, Newell W, Mata P, Myatt G, Sike S, Zsoldos Z, Johnson AP (1994). SPROUT: recent developments in the de novo design of molecules. J Chem Inf Model.

[9] Pearlman DA, Murcko MA (1996). CONCERTS: dynamic connection of fragments as an approach to de novo ligand design. J Med Chem.

[10] Langdon SR, Ertl P, Brown N (2010). Bioisosteric replacement and scaffold hopping in lead generation and optimization. Mol Inf.

[11] Sun H, Tawa G, Wallqvist A (2012). Classification of scaffold-hopping approaches. Drug Discovery Today.

[12] Schneider G, Lee M-L, Stahl M, Schneider P (2000). De novo design of molecular architectures by evolutionary assembly of drug-derived building blocks. J Comput Aided Mol Des.

[13] Vinkers HM, de Jonge MR, Daeyaert FF, Heeres J, Koymans LM, van Lenthe JH, Lewi PJ, Timmerman H, Van Aken K, Janssen PA (2003). Synopsis: synthesize and optimize system in silico. J Med Chem.

[14] Hartenfeller M, Zettl H, Walter M, Rupp M, Reisen F, Proschak E, Weggen S, Stark H, Schneider G (2012). DOGS: Reaction-Driven de novo Design of Bioactive Compounds. PLoS Comput Biol.

[15] Nishibata Y, Itai A (1991). Automatic creation of drug candidate structures based on receptor structure. Starting point for artificial lead generation. Tetrahedron.

[16] Böhm H-J (1992). LUDI: rule-based automatic design of new substituents for enzyme inhibitor leads. J Comput-Aided Mol Des.

[17] Clark DE, Frenkel D, Levy SA, Li J, Murray CW, Robson B, Waszkowycz B, Westhead DR (1995). PRO_LIGAND: An approach to de novo molecular design .1. Application to the design of organic molecules. J Comput-Aided Mol Des.

[18] Gillet VJ, Newell W, Mata P, Myatt G, Sike S, Zsoldos Z, Johnson AP (1994). SPROUT: recent developments in the de novo design of molecules. J Chem Inf Comput Sci.

[19] Agarwal AK, Johnson AP, Fishwick CW (2008). Synthesis of de novo designed small-molecule inhibitors of bacterial RNA polymerase. Tetrahedron.

[20] Ji H, Li H, Martásek P, Roman LJ, Poulos TL, Silverman RB (2009). Discovery of highly potent and selective inhibitors of neuronal nitric oxide synthase by fragment hopping. J Med Chem.

[21] Ji H, Stanton BZ, Igarashi J, Li H, Martásek P, Roman LJ, Poulos TL, Silverman RB (2008). Minimal pharmacophoric elements and fragment hopping, an approach directed at molecular diversity and isozyme selectivity. Design of selective neuronal nitric oxide synthase inhibitors. J Am Chem Soc.

[22] Sova M, Čadež G, Turk S, Majce V, Polanc S, Batson S, Lloyd AJ, Roper DI, Fishwick CW, Gobec S (2009). Design and synthesis of new hydroxyethylamines as inhibitors of D-alanyl-D-lactate ligase (VanA) and D-alanyl-D-alanine ligase (DdlB). Bioorg Med Chem Lett.

[23] Pierce AC, Rao G, Bemis GW (2004). BREED: Generating novel inhibitors through hybridization of known ligands. Application to CDK2, P38, and HIV protease. J Med Chem.

[24] Schneider G, Lee M-L, Stahl M, Schneider P (2000). De novo design of molecular architectures by evolutionary assembly of drug-derived building blocks. J Comput-Aided Mol Des.

[25] Kutchukian PS, Shakhnovich EI (2010). De novo design: balancing novelty and confined chemical space. Expert Opin Drug Discov.

[26] Lewell XQ, Judd DB, Watson SP, Hann MM (1998). Recap retrosynthetic combinatorial analysis procedure: a powerful new technique for identifying privileged molecular fragments with useful applications in combinatorial chemistry. J Chem Inf Comput Sci.

[27] Sanchez-Lengeling B, Aspuru-Guzik A (2018). Inverse molecular design using machine learning: Generative models for matter engineering. Science.

[28] Cheng Y, Gong Y, Liu Y, Song B, Zou Q (2021). Molecular design in drug discovery: a comprehensive review of deep generative models. Briefings Bioinf.

[29] Gupta A, Mueller AT, Huisman BJH, Fuchs JA, Schneider P, Schneider G (2018). Generative Recurrent Networks for De Novo Drug Design. Mol Inf.

[30] Moret M, Friedrich L, Grisoni F, Merk D, Schneider G (2020). Generative molecular design in low data regimes. Nat Mach Intell.

[31] Segler MHS, Kogej T, Tyrchan C, Waller MP (2018). Generating Focused Molecule Libraries for Drug Discovery with Recurrent Neural Networks. ACS Cent Sci.

[32] Santana MVS, Silva-Jr FP (2021). De novo design and bioactivity prediction of SARS-CoV-2 main protease inhibitors using recurrent neural network-based transfer learning. BMC Chem.

[33] Gomez-Bombarelli R, Wei JN, Duvenaud D, Hernandez-Lobato JM, Sanchez-Lengeling B, Sheberla D, Aguilera-Iparraguirre J, Hirzel TD, Adams RP, Aspuru-Guzik A (2018). Automatic Chemical Design Using a Data-Driven Continuous Representation of Molecules. ACS Cent Sci.

[34] Skalic M, Jiménez J, Sabbadin D, De Fabritiis G (2019). Shape-based generative modeling for de novo drug design. J Chem Inf Model.

[35] Zhavoronkov A, Ivanenkov YA, Aliper A, Veselov MS, Aladinskiy VA, Aladinskaya AV, Terentiev VA, Polykovskiy DA, Kuznetsov MD, Asadulaev A (2019). Deep learning enables rapid identification of potent DDR1 kinase inhibitors. Nat Biotechnol.

[36] Kadurin A, Nikolenko S, Khrabrov K, Aliper A, Zhavoronkov A (2017). druGAN: an advanced generative adversarial autoencoder model for de novo generation of new molecules with desired molecular properties in silico. Mol Pharmaceutics.

[37] Guimaraes GL, Sanchez-Lengeling B, Outeiral C, Farias PLC, Aspuru-Guzik A: Objective-Reinforced Generative Adversarial Networks (ORGAN) for Sequence Generation Models. arXiv 2018:1705.10843.

[38] Sanchez-Lengeling B, Outeiral C, Guimaraes GL, Aspuru-Guzik A: Optimizing distributions over molecular space. An Objective-Reinforced Generative Adversarial Network for Inverse-design Chemistry (ORGANIC). chemrxiv 2017:5309668.

[39] Wang J, Hsieh C-Y, Wang M, Wang X, Wu Z, Jiang D, Liao B, Zhang X, Yang B, He Q (2021). Multi-constraint molecular generation based on conditional transformer, knowledge distillation and reinforcement learning. Nat Mach Intell.

[40] Skalic M, Sabbadin D, Sattarov B, Sciabola S, De Fabritiis G (2019). From Target to Drug: Generative Modeling for the Multimodal Structure-Based Ligand Design. Mol Pharmaceutics.

[41] Bai Q, Tan S, Xu T, Liu H, Huang J, Yao X (2021). MolAICal: a soft tool for 3D drug design of protein targets by artificial intelligence and classical algorithm. Briefings Bioinf.

[42] Xu MY, Ran T, Chen HM (2021). De novo molecule design through the molecular generative model conditioned by 3D Information of Protein Binding Sites. J Chem Inf Model.

[43] Li Y, Pei J, Lai L (2021). Structure-based de novo drug design using 3D deep generative models. Chem Sci.

[44] Ragoza M, Masuda T, Koes DR (2022). Generating 3D molecules conditional on receptor binding sites with deep generative models. Chem Sci.

[45] Joshi RP, Gebauer NW, Bontha M, Khazaieli M, James RM, Brown JB, Kumar N (2021). 3D-Scaffold: A Deep Learning Framework to Generate 3D Coordinates of Drug-like Molecules with Desired Scaffolds. J Phys Chem B.

[46] Gebauer NW, Gastegger M, Hessmann SS, Müller K-R, Schütt KT (2022). Inverse design of 3d molecular structures with conditional generative neural networks. Nat Commun.

[47] Gebauer N, Gastegger M, Schütt K: Symmetry-adapted generation of 3d point sets for the targeted discovery of molecules. Advances in Neural Information Processing Systems 2019, 32.

[48] Imrie F, Hadfield TE, Bradley AR, Deane CM (2021). Deep generative design with 3D pharmacophoric constraints. Chem Sci.

[49] Wang M, Hsieh C-Y, Wang J, Wang D, Weng G, Shen C, Yao X, Bing Z, Li H, Cao D (2022). RELATION: A Deep Generative Model for Structure-Based De Novo Drug Design. J Med Chem.

[50] Sun B, Feng J, Saenko K: Return of frustratingly easy domain adaptation. In: Proceedings of the AAAI Conference on Artificial Intelligence: 2016.

[51] Tzeng E, Hoffman J, Darrell T, Saenko K: Simultaneous deep transfer across domains and tasks. In: Proceedings of the IEEE international conference on computer vision: 2015. 4068–4076.

[52] Cai C, Wang S, Xu Y, Zhang W, Tang K, Ouyang Q, Lai L, Pei J (2020). Transfer Learning for Drug Discovery. J Med Chem.

[53] Taylor ME, Stone P (2009). Transfer Learning for Reinforcement Learning Domains: A Survey. J Mach Learn Res.

[54] Bousmalis K, Trigeorgis G, Silberman N, Krishnan D, Erhan D (2016). Domain separation networks arXiv.

[55] Eberhardt J, Santos-Martins D, Tillack AF, Forli S: AutoDock Vina 1.2.0: New Docking Methods, Expanded Force Field, and Python Bindings. J Chem Inf Model 2021, 61:3891–3898. 10.1021/acs.jcim.1c00203 PMC10683950 34278794

[56] Griffiths R-R, Hernandez-Lobato JM (2020). Constrained Bayesian optimization for automatic chemical design using variational autoencoders. Chem Sci.

[57] Wolber G, Langer T (2005). LigandScout: 3-d pharmacophores derived from protein-bound Ligands and their use as virtual screening filters. J Chem Inf Model.

[58] Wolber G, Dornhofer AA, Langer T (2006). Efficient overlay of small organic molecules using 3D pharmacophores. J Comput-Aided Mol Des.

[59] Irwin JJ, Sterling T, Mysinger MM, Bolstad ES, Coleman RG (2012). ZINC: A Free Tool to Discover Chemistry for Biology. J Chem Inf Model.

[60] Sterling T, Irwin JJ (2015). ZINC 15 – Ligand Discovery for Everyone. J Chem Inf Model.

[61] Gilson MK, Liu T, Baitaluk M, Nicola G, Hwang L, Chong J (2016). BindingDB in 2015: A public database for medicinal chemistry, computational chemistry and systems pharmacology. Nucleic Acids Res.

[62] Davies M, Nowotka M, Papadatos G, Dedman N, Gaulton A, Atkinson F, Bellis L, Overington JP (2015). ChEMBL web services: streamlining access to drug discovery data and utilities. Nucleic Acids Res.

[63] Berman HM, Westbrook J, Feng Z, Gilliland G, Bhat TN, Weissig H, Shindyalov IN, Bourne PE (2000). The Protein Data Bank. Nucleic Acids Res.

[64] Otasek D, Morris JH, Boucas J, Pico AR, Demchak B (2019). Cytoscape Automation: empowering workflow-based network analysis. Genome Biol.

[65] Saito R, Smoot ME, Ono K, Ruscheinski J, Wang P-L, Lotia S, Pico AR, Bader GD, Ideker T (2012). A travel guide to Cytoscape plugins. Nat Methods.

[66] Su X, Hu L, You Z, Hu P, Wang L, Zhao B: A deep learning method for repurposing antiviral drugs against new viruses via multi-view nonnegative matrix factorization and its application to SARS-CoV-2. Briefings Bioinf 2021, 23:bbab526. 10.1093/bib/bbab526 34965582

[67] Su X, Hu L, You Z, Hu P, Zhao B: Attention-based Knowledge Graph Representation Learning for Predicting Drug-drug Interactions. Briefings Bioinf 2022, 23:bbac140. 10.1093/bib/bbac140 35453147

[68] Chen X, Liu M-X, Yan G-Y (2012). Drug-target interaction prediction by random walk on the heterogeneous network. Mol BioSyst.

[69] Zhao T, Hu Y, Valsdottir LR, Zang T, Peng J (2021). Identifying drug-target interactions based on graph convolutional network and deep neural network. Briefings Bioinf.

[70] Steuer CE, Khuri FR, Ramalingam SS (2015). The next generation of epidermal growth factor receptor tyrosine kinase inhibitors in the treatment of lung cancer. Cancer.

