# Supplementary material for: ReMODE: a deep learning-based web server for target-specific drug design
Source: J Cheminform. 2022 Dec 12;14:84. doi: 10.1186/s13321-022-00665-w (PMC9743675; doi:10.1186/s13321-022-00665-w)
Supplement: Supplementary file 1 — Additional file 1. [file 13321_2022_665_MOESM1_ESM.docx]

**SUPPLEMENTARY DATA**

# ReMODE: a deep learning-based web server for target-specific drug design

Mingyang Wang^1,2,#^, Jike Wang^1,2,#^, Gaoqi Weng^1,2^, Yu Kang^1^, Peichen Pan^1^, Dan Li^1^, Yafeng Deng^2^, Honglin Li^3,*^, Chang-Yu Hsieh^1,*^, Tingjun Hou^1,*^

^1^Innovation Institute for Artificial Intelligence in Medicine of Zhejiang University, College of Pharmaceutical Sciences and Cancer Center, Zhejiang University, Hangzhou 310058, Zhejiang, P. R. China

^2^CarbonSilicon AI Technology Co., Ltd, Hangzhou 310018, Zhejiang, P. R. China

^3^Shanghai Key Laboratory of New Drug Design, School of Pharmacy, East China University of Science & Technology, Shanghai 200237, P. R. China

^#^These authors contribute equally

Corresponding authors

**Honglin Li: E-mail**: hlli@ecust.edu.cn

**Chang-Yu Hsieh: E-mail**: kimhsieh@tencent.com

**Tingjun Hou: E-mail**: tingjunhou@zju.edu.cn

**CONTENT**

[The algorithms of Uncon. Gen. and Bo. Gen. 3](#_Toc108185943)

[The algorithm of Prop. gen. 3](#_Toc108185944)

[The algorithm of Smi. gen. 3](#_Toc108185945)

[Evaluation metrics in result page 3](#_Toc108185946)

[Figure S1 5](#_Toc108185947)

[Figure S2. 6](#_Toc108185948)

[Figure S3 7](#_Toc108185949)

[Figure S4 8](#_Toc108185950)

[Figure S5. 9](#_Toc108185951)

[Table S1 10](#_Toc108185952)

[Table S2 11](#_Toc108185953)

[Table S3 12](#_Toc108185954)

[Abbreviations 13](#_Toc108185955)

[References 13](#_Toc108185956)

# The algorithms of Uncon. Gen. and Bo. Gen.

The algorithm of unconditional generation and Bayesian sampling without the ‘optimization’ module is consistent with that used in RELATION reported in our previous study [[1](#_ENREF_1)]. The loss function in the training process and network parameters were recorded in the **Table S1** and **Table S2**, and the Bayesian sampling method was recorded in **Table S3**.

# The algorithm of Prop. gen.

In the ‘Physicochemical properties’ module, the CVAE architecture in ReMODE directly produces molecules with the customized target properties. The key difference of CVAE-ReMODE from the original ReMODE is to embed the conditional information into the objective function of the VAE loss in **Table S1**, leading to the revised objective function as follow:

$$\mathcal{L}_{latent-cvae}={-D}_{KL}\left( q\left( H|(X,c) \right)\|N\left( 0,I \right) \right)$$

where $c$ denotes a condition vector. The condition vector $c$ is directly involved in the encoding and decoding processes. In our model, the physicochemical properties MW, logP, QED and SA were represented as the condition vector. As a result, the CVAE in the ‘Physicochemical properties’ module can generate molecules with the target properties imposed by the condition vector.

# The algorithm of Smi. gen.

When exploring the latent space of VAE, the Euclidean distance between the vectors map directly to the scaffold similarity of molecules [[2](#_ENREF_2)]. In the fragment-based generation module, the network of unconditional generation was selected as the basic architecture. Then, the input molecule (fragment/scaffold) will be decoded into the latent space as an anchor vector. Finally, a certain number (the number of generated molecules defined by users) of vectors are randomly sampled within a Euclidean distance of 5 around the anchor vector and decoded into a molecule set for users.

# Evaluation metrics

The metrics (**formula 1~3**) in our study and server were used to measure the quality of the baseline molecule dataset (*G*) against the existing (or training) dataset (*E*):

$$\begin{aligned} G_{validity}=\frac{\left| V_{G} \right|}{N_{G}} \#(1) \end{aligned}$$

$$\begin{aligned} G_{novel}=1-\frac{\left| set\left( V_{G}\cap E \right) \right|}{V_{G}} \#(2) \end{aligned}$$

$$\begin{aligned} G_{uniqueness}=\frac{\left| set\left( V_{G} \right) \right|}{\left| V_{G} \right|}\#(3) \end{aligned}$$

$$\begin{aligned} G_{internal diversity}=1-\frac{1}{\left| set\left( V \right) \right|^{2}}\sum_{\left( a,b \right)\in set\left( V \right)} T\left( a,b \right) \#(4) \end{aligned}$$

*Validity* (**formula 1**) is used to evaluate the validity rate of the SMILES strings in *G*, where $N_{G}$ is the number of generated molecules and $V_{E}$ is the valid SMILES strings in *G*. *Novelty* (**formula 2**) is used to evaluate the proportion of compounds that exist in *G* but not in *E*. Internal Diversity is used to evaluate the diversity of compounds in *G*, in which the last term in **formula 4** calculates the average Tanimoto coefficient ($T$) between the generated molecules.

The calculation of *FCD* (**formula 5**) is firstly to take the information of the compound bioactivity in the penultimate layer of ChemNet [[3](#_ENREF_3)], in which the Gaussian distribution vector includes the structure information and bioactivity properties of the compounds, and then can be defined as:

$$\begin{aligned} FCD\left( G,E \right)=\left\| m_{G}-m_{E} \right\|_{2}^{2}+Tr\left( \Sigma_{G}+\Sigma_{R}{-2\left( \Sigma_{G}\Sigma_{E} \right)}^{\frac{1}{2}} \right) \#(5) \end{aligned}$$

where $m_{G}$ and $m_{E}$ are the mean vectors and $\Sigma_{G}$ and $\Sigma_{R}$ are the covariance matrices of the activations on the penultimate layer of ChemNet on the datasets G and E, respectively.

The calculation of pharmacophore and AutoDock Vina scores can be referenced to our previous study [[1](#_ENREF_1)].


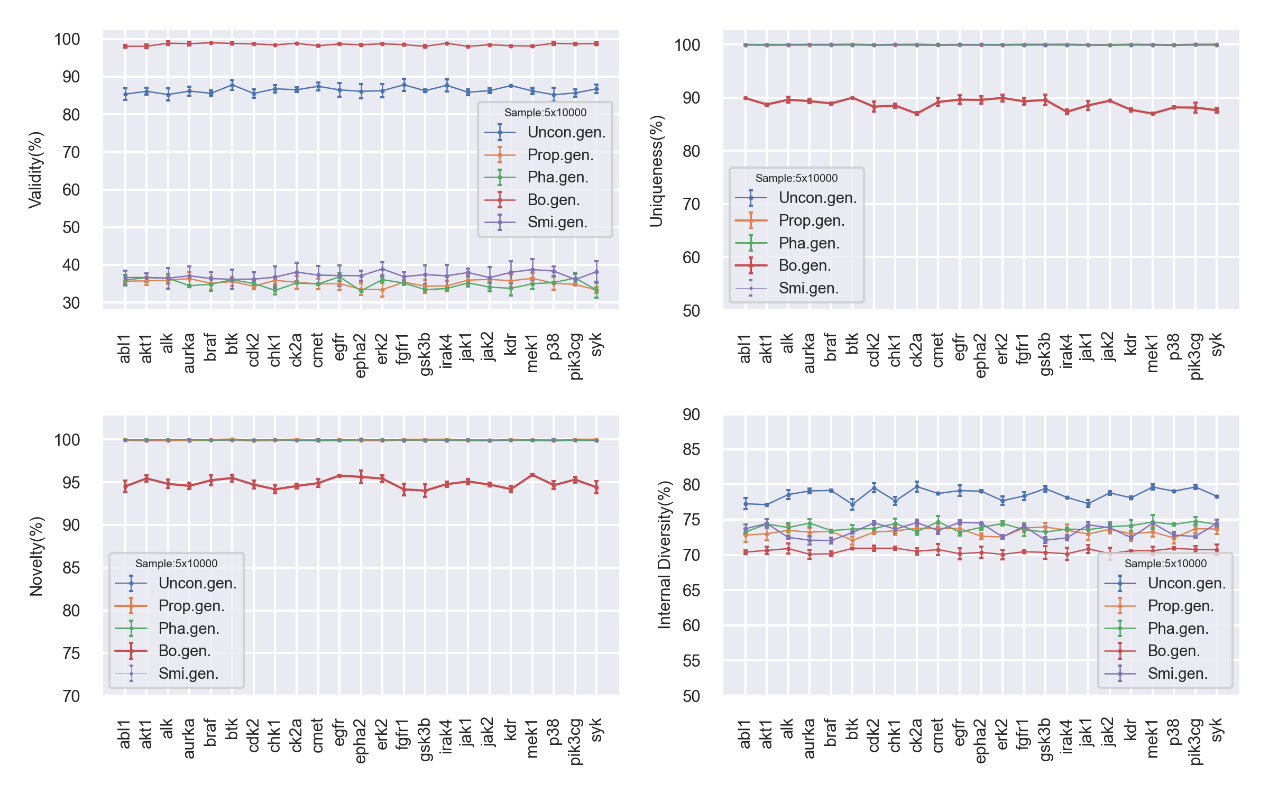


# Figure S1. Validity, uniqueness, novelty and internal diversity of the 10,000 generated molecules for the target-specific task of all the targets in the list. The top curve in Uniqueness and Novelty are actually a combination of multiple curves with values close to 100%.


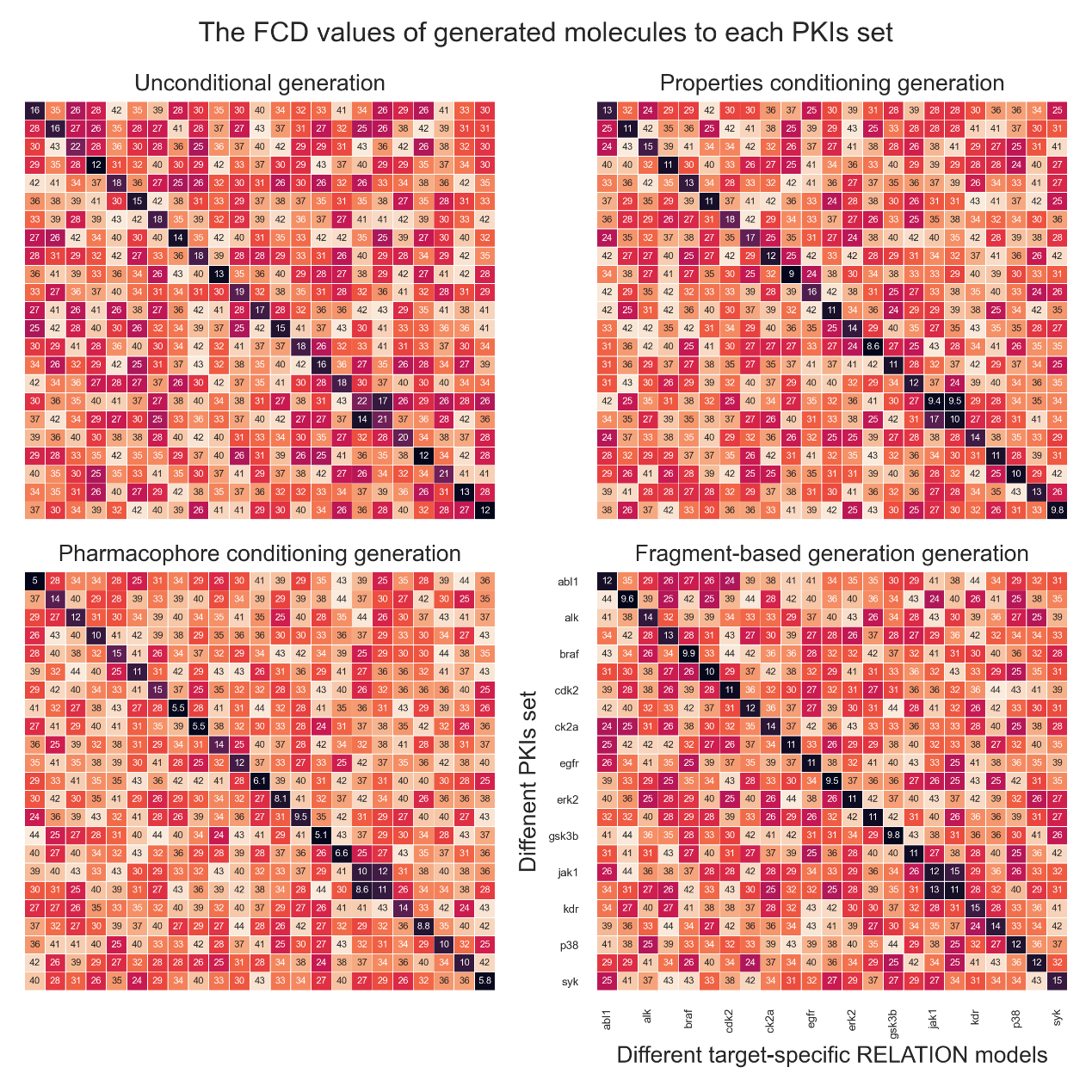


# Figure S2. The FCD value of the 10,000 valid molecules generated by different modules for all the 23 protein targets in the list.


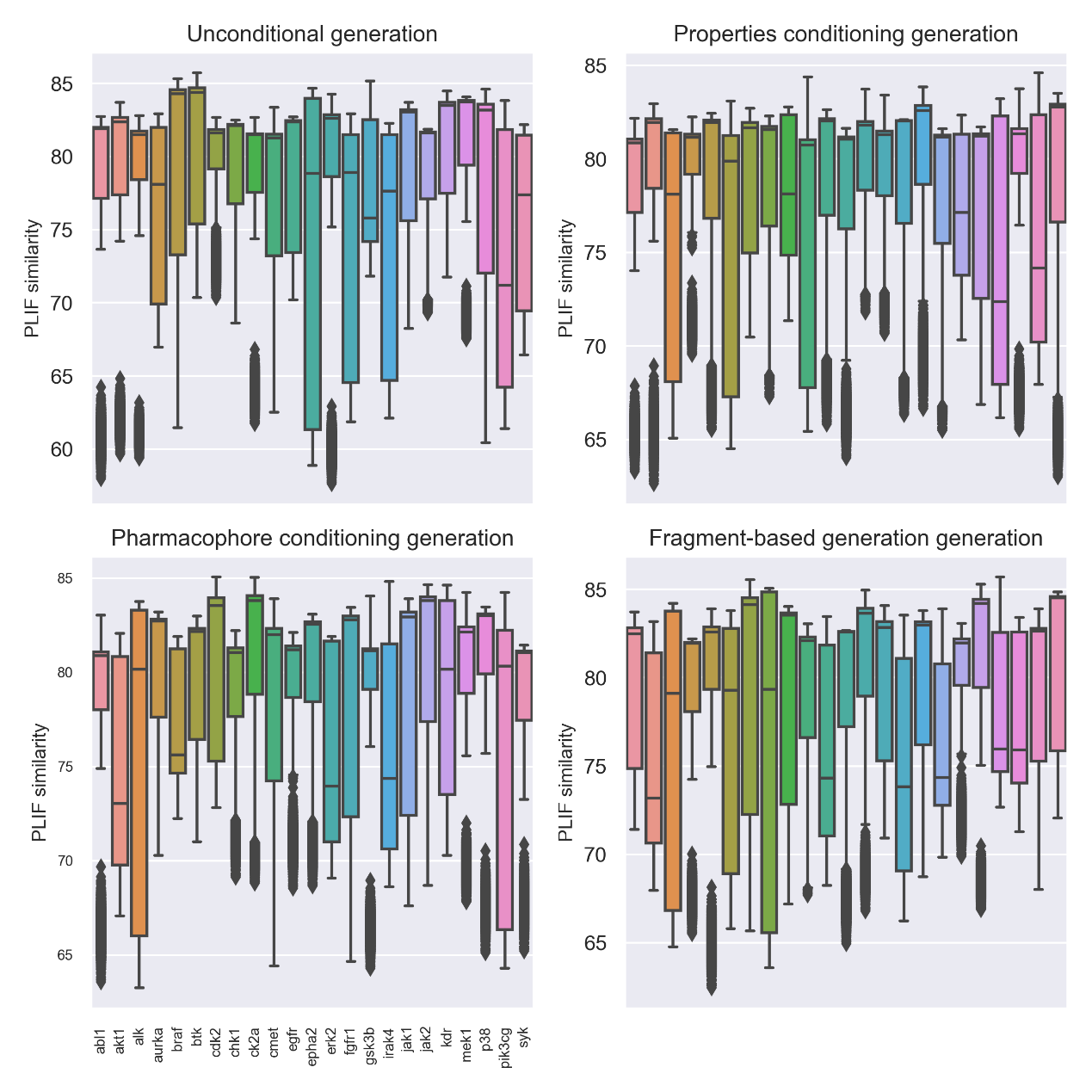


# Figure S3. Average SNN of PLIF between the 10,000 valid molecules generated by different modules based on the PKIs set.


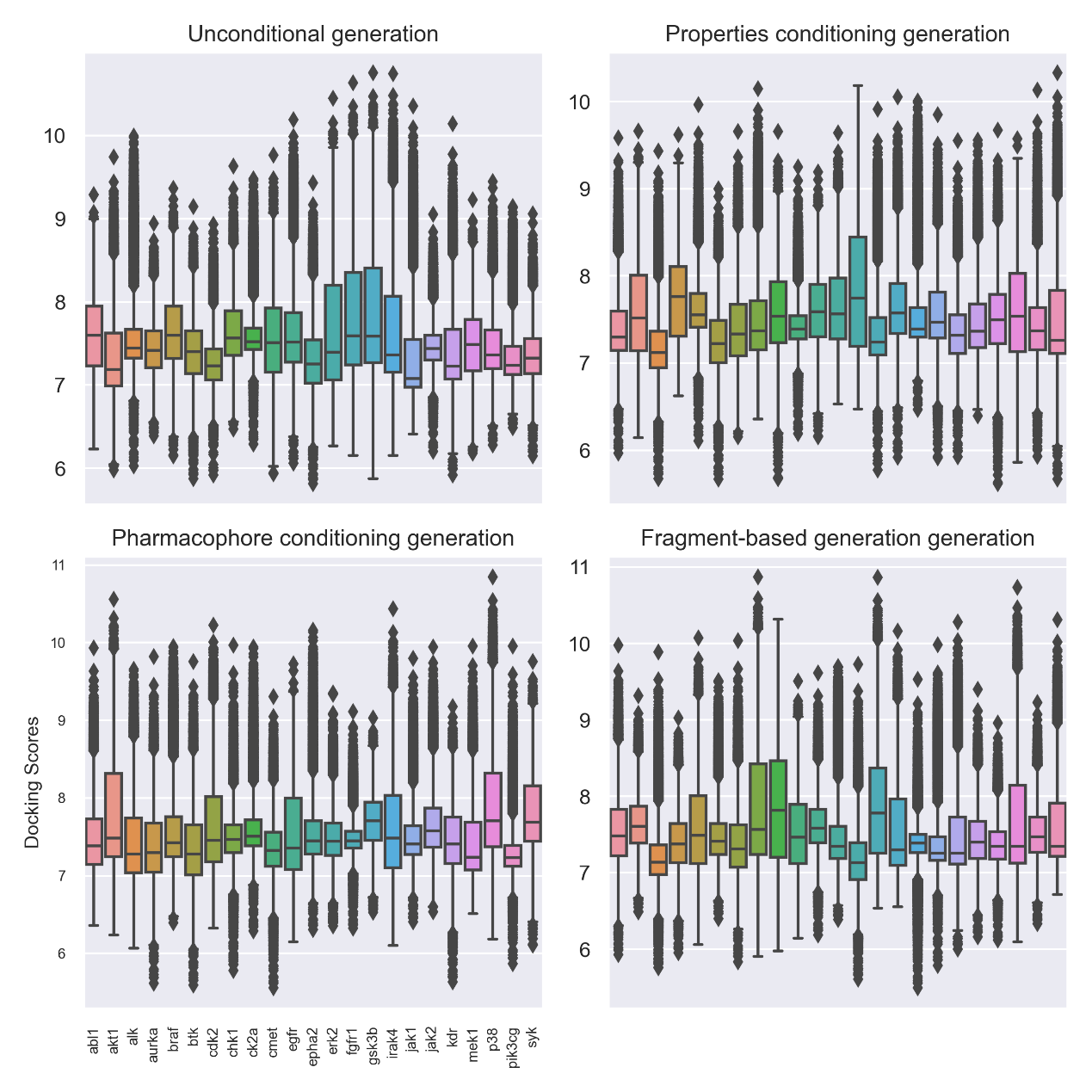


# Figure S4. Docking score distribution of the 10,000 valid molecules generated by different modules based on the PKIs set.


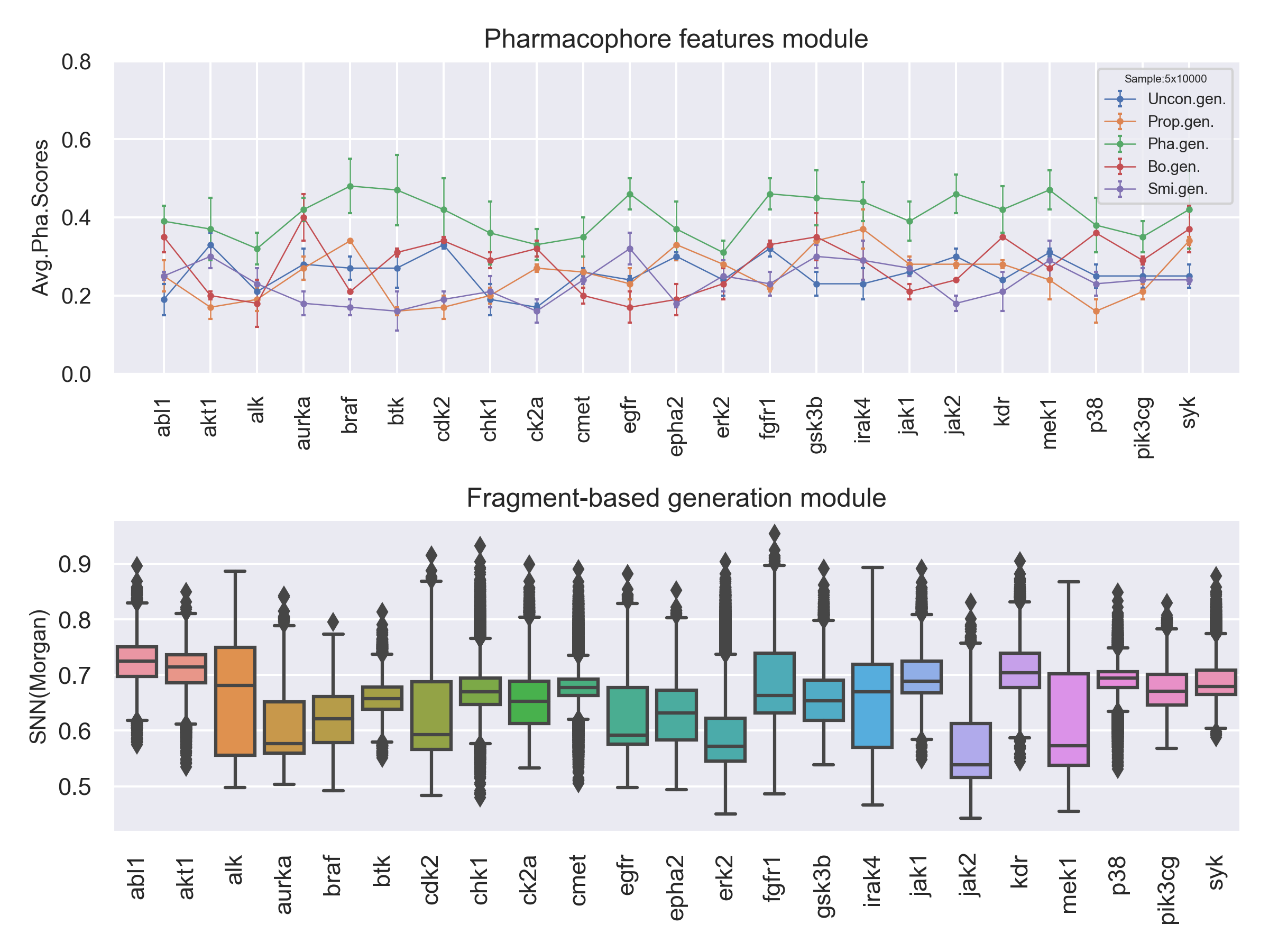


# Figure S5. Performance of the ‘Pharmacophore features’ and ‘Structure features’ modules: (A) The average pharmacophore scores (n=5) of the 10,000 valid molecules generated by different modules; (B) The SNN (Morgan) distribution of the 10,000 valid molecules generated by different modules to the input SMILES strings, and the input SMILES was randomly selected from the PKIs set of the selected target.

# Table S1. The loss function in the training process.

| Loss function | Annotation |
| --- | --- |
| $\mathcal{L}_{\boldsymbol{latent}}^{\boldsymbol{1}}\boldsymbol{=}\boldsymbol{-D}_{\boldsymbol{KL}}\left( \boldsymbol{q}\left( \boldsymbol{H\vert X} \right)\boldsymbol{\Vert N}\left( \boldsymbol{0,I} \right) \right)$ | $q\left( H\vert X \right)$ refers to probabilistic encoding distribution. ${-D}_{KL}$ was applied to calculate the Kullback-Leibler divergence [[4](#_ENREF_4)] between the prior and the Gaussian distribution. |
| $\begin{aligned} \mathcal{L}_{\boldsymbol{diff}}^{\boldsymbol{2}}\boldsymbol{=}\left\Vert{{\boldsymbol{H}_{\boldsymbol{pri}}^{\boldsymbol{com}}}^{\boldsymbol{┬}}\boldsymbol{H}}_{\boldsymbol{shr}}^{\boldsymbol{com}} \right\Vert_{\boldsymbol{F}}^{\boldsymbol{2}}\boldsymbol{+}\left\Vert{{\boldsymbol{H}_{\boldsymbol{pri}}^{\boldsymbol{lig}}}^{\boldsymbol{┬}}\boldsymbol{H}}_{\boldsymbol{shr}}^{\boldsymbol{lig}} \right\Vert_{\boldsymbol{F}}^{\boldsymbol{2}} \end{aligned}$ | $\left\Vert\cdot\right\Vert_{F}^{2}$ refers to the squared Frobenius norm [[5](#_ENREF_5)] between private and share latent code. |
| $\mathcal{L}_{\boldsymbol{sim}}^{\boldsymbol{3}}\boldsymbol{=}{\boldsymbol{MMD}\left( \boldsymbol{H}_{\boldsymbol{shr}}^{\boldsymbol{com}}\boldsymbol{,}\boldsymbol{H}_{\boldsymbol{shr}}^{\boldsymbol{lig}} \right)}^{\boldsymbol{2}}$ | $MMD$ refer to Maximum Mean Discrepancy [[6](#_ENREF_6)]. |
| $\begin{aligned} \mathcal{L}_{\boldsymbol{caption}}^{\boldsymbol{4}}\boldsymbol{=-}\frac{\boldsymbol{1}}{\boldsymbol{N}}\sum_{\boldsymbol{i=1}}^{\boldsymbol{N}^{\boldsymbol{com}}} \sum_{\boldsymbol{j=1}}^{\boldsymbol{length}} \boldsymbol{lig}_{\boldsymbol{ij}}\boldsymbol{log(}{\hat{\boldsymbol{lig}}}_{\boldsymbol{ij}}\boldsymbol{)} \end{aligned}$ | ${N, lig}_{i}$, $\hat{lig}_{i}$ and *length* represent the batch size, ground-truth, the grids of the generated molecules and the generated molecules, respectively. |

^1^The latent loss of VAE; ^2^The different loss, which can ensure that the generated molecules have the characteristics of source data (ligand) and target data (ligand-protein), $H_{pri}^{com}{,H}_{pri}^{lig}$: the private encoder of target and source data, $H_{shr}^{com}{,H}_{shr}^{lig}$: the shared encoder of target and source data; ^3^The similar loss is used to catch the similarity between the source data and target data; ^4^The caption loss is used to generated the molecular strings in latent space.

# Table S2. Hyperparameters available in the ReMODE network.

| Hyper parameters | Values |
| --- | --- |
| Learning rate | 0.01 |
| Epochs | 100 or 150 |
| Batch size | 32 |
| Activation function | ReLU |
| Batch normalization | Yes |
| Weight of Loss function | α=0.01, β=0.075, γ=0.25 |
| Vocabulary size | 39 |
| Encoder (3D-CNN) | 8 layers: 64, 64, 128, 128, 256, 256,  512, 512 |
| Decoder (Caption-LSTM) | 3 layers |
| Hidden size of decoder | 1024 |

# Table S3. Bayesian Optimization procedure.

| Algorithm 1: Bayesian Optimization |
| --- |
| **Input**: black-box objective vina docking scores function $f(x)$, acquisition function $\alpha$, surrogate model $\hat{f}(x)$, latent molecular point $\mathcal{X}$  **Select** random batch $S\subset\mathcal{X}$  **Initialize** $\mathcal{D\leftarrow}\left\{ \left( x,f\left( x \right) \right):x\in S \right\}$  **for** $t\leftarrow1$ to $T$ do  Train surrogate model $\hat{f}(x)$ using $\mathcal{D}$  Select batch $S\leftarrow{}_{\mathcal{B\subset X}}^{argmax}{}\sum_{x\mathcal{\in B}} \alpha(x,\hat{f},f^{*})$  Update $\mathcal{D\leftarrow D\cup}\left\{ \left( x,f\left( x \right) \right): x\in S \right\}$ |
| **end**  **Result:**${\{x_{i}\}}_{i=1}^{k}={}_{{\{x_{i}\}}_{i=1}^{k *}\mathcal{\subset D}}^{argmax}{}\sum_{i=1}^{k} f(x_{i})$ |

^*^$f\left( x \right)$ is a black-box objective function, sparse GP was used for modelling the surrogate model $\hat{f}(x)$, expected improvement (EI) was used as acquisition function $\alpha$.

Abbreviations

Uncon.gen.: Unconditional generation without ‘optimization’ module; Prop.gen.: Properties features generation in ‘Physicochemical properties’ module; Bo.gen.: Bayesian optimization generation in ‘Bayesian sampling’ module; Pha.gen.: Pharmacophore constraints generation in ‘Pharmacophore features’; CVAE: Conditional variational autoencoder; Smi.gen.: Fragment-based generation in ‘Structure features’ modules; MW: Molecular weight; logP: Wildman-Crippen partition coefficient; QED: Quantitative estimate of drug-likeness; SA: synthesis accessibility; FCD: Fréchet ChemNet Distance

# References

1. Wang M, Hsieh C-Y, Wang J, Wang D, Weng G, Shen C, Yao X, Bing Z, Li H, Cao D *et al*: RELATION: A Deep Generative Model for Structure-Based De Novo Drug Design. J Med Chem 2022, 65:9478-9492.

2. Prasad KS, Chander OS, Reddy GP, Gururaj S: Artificial Intelligence approach for Classifying Molecular Dataset using Density based technique with appropriate Euclidean Distance measure. In: International Conference on Advancements in Aeromechanical Materials for Manufacturing (ICAAMM): 2017; MLR Inst Technol, Hyderabad, INDIA. 2017: 8827-8836.

3. Preuer K, Renz P, Unterthiner T, Hochreiter S, Klambauer G: Frechet ChemNet Distance: A Metric for Generative Models for Molecules in Drug Discovery. J Chem Inf Model 2018, 58:1736-1741.

4. Hershey JR, Olsen PA: Approximating the Kullback Leibler divergence between Gaussian mixture models. In: 2007 IEEE International Conference on Acoustics, Speech and Signal Processing-ICASSP'07. IEEE 2007: IV317-IV320.

5. Böttcher A, Wenzel D: How big can the commutator of two matrices be and how big is it typically? Linear Algebra Appl 2005, 403:216-228.

6. Gretton A, Borgwardt KM, Rasch MJ, Schölkopf B, Smola A: A kernel two-sample test. J Mach Learn Res 2012, 13:723-773.
